# Private health insurance coverage of drug use disorder treatment: 2005–2018

**DOI:** 10.1371/journal.pone.0240298

**Published:** 2020-10-09

**Authors:** Ramin Mojtabai, Christine Mauro, Melanie M. Wall, Colleen L. Barry, Mark Olfson

**Affiliations:** 1 Department of Mental Health, Bloomberg School of Public Health and Department of Psychiatry, Johns Hopkins University, Baltimore, MD, United States of America; 2 Mailman School of Public Health, Columbia University, New York, NY, United States of America; 3 Department of Psychiatry, Vagelos College of Physicians and Surgeons, Columbia University, New York, NY, United States of America; 4 Department of Health Policy and Management and Department of Mental Health, Bloomberg School of Public Health, Johns Hopkins University, Baltimore, MD, United States of America; University of Arkansas for Medical Sciences, UNITED STATES

## Abstract

Many privately insured adults with drug use disorders in the United States do not have health care coverage for drug use treatment. The Affordable Care Act sought to redress this gap by including substance use treatments as essential health benefits under new plans offered. This study used data from 11,732 privately insured adult participants of the 2005–2018 National Survey on Drug Use and Health with drug use disorders to examine trends in drug use treatment coverage and the association of coverage with receiving treatment. 37.6% of the participants with drug use disorders did not know whether their plan covered drug use treatment, with little change over time. Among those who knew, coverage increased modestly between the 2005–2013 and 2014–2018 periods (73.5% vs. 77.5%, respectively, *p* = .015). Coverage was associated with receiving drug use treatment (adjusted odds ratio = 2.09, 95% confidence interval = 1.61–2.72, *p* < .001). However, even among participants with coverage, only 13.4% received treatment. Broader coverage of drug use treatment could potentially improve treatment rates.

## Introduction

The Paul Wellstone and Pete Domenici Mental Health Parity and Addiction Equity Act (MHPAEA) of 2008 sought to eliminate historical differences in insurance coverage for substance use and mental health treatment services compared with physical health services in private health insurance plans [1–4]. However, this law only applied to health plans offered by firms with fifty or more employees choosing to offer these services in their benefit packages. Plans that did not cover treatment of drugs and alcohol use disorders or mental health conditions as a part of their benefit packages were not subject to the law [1].

Beginning in 2014, the Affordable Care Act (ACA) expanded the reach of MHPAEA by mandating coverage of substance use and mental health insurance benefits as part of the essential health benefits requirement for insurance plans sold in the individual and small group markets [5]. Although there are wide variations among states in the type and extent of services covered under the 10 essential health benefits requirements [6], early research findings indicate an increase in coverage of mental health and substance use treatment services following implementation of this policy [7]. In an analysis of commercial plans offered in individual and small-group markets in 12 states, coverage of inpatient, outpatient and office-based substance use treatment increased from 77% in 2013 to 100% in 2014 [7]. However, many existing large group commercial plans were exempted from the essential health benefit requirement (“grandfathered”) [8]. As late as 2017, 17% of workers nationally were covered by such grandfathered plans [8].

While little is known about changes in rates of coverage of substance use treatment services in private insurance plans following implementation of ACA, there is some evidence of increased use of mental and substance use treatment services in the post-ACA period. Much of research on the impact of ACA on mental health and substance use services, however, has focused on Medicaid expansion [9–16]. This focus is appropriate because Medicaid is a major source of funding for mental health and substance use treatment and a large proportion of adults with these disorders qualify for Medicaid. Comparatively less research has examined the effect of changes in private health plans on use of behavioral health services [7,17]. However, the majority of adults in the United States (US) are covered by private health insurance plans and the socio-demographic and service use profiles of individuals covered by private health insurance differ significantly from those covered by Medicaid [18]. As such, research from Medicaid-covered populations may not directly generalize to privately insured people.

The present study seeks to fill a gap in knowledge regarding changes in coverage of behavioral health services under private health insurance plans in the years following implementation of ACA and parity legislation. Furthermore, this study focuses on drug use treatment coverage because private insurance plans have been more likely to exclude coverage for these services relative to other behavioral health services in the years before implementation of ACA [19]. Specifically, the present study uses data from the National Survey on Drug Use and Health (NSDUH) for years 2005–2018 to examine trends in coverage of drug use treatment among privately insured individuals with drug use disorders. The study also examines the association of such coverage with actual treatment for drug use disorders. An earlier study using NSDUH data examined association of health insurance, including private health insurance, with receipt of substance use treatment [16]. However, that study covered years 2005–2009—the period before implementation of MHPAEA and ACA—and examined association of insurance coverage with treatment seeking for either alcohol or drug use disorder. The present study extends the time to 2018 and specifically examines the association of coverage with drug use treatment.

## Materials and methods

### Study population

The NSDUH survey methods have been well described elsewhere [20]. In brief, NSDUH is a national household survey conducted annually by the Substance Abuse and Mental Health Services Administration (SAMHSA) through face-to-face interviews with sampled adults and adolescents. The present study focuses on individuals aged 18 years and older. A stratified, multistage area probability sampling design was used with states as the primary strata and state sampling regions serving as the secondary strata. Census tracts, census block groups, segments within census block groups, and dwelling units within segments were selected using probability-proportional-to-size sampling. After selecting dwelling units, an interviewer visited each unit to obtain a roster of household residents. Up to two residents who were 12 years old or older were selected for interviewing based on this roster. Persons without a household address (e.g., homeless persons not in shelters), active-duty military, and institutional residents were not included. NSDUH oversamples adolescents and young adults. However, the weighted NSDUH sample is representative of the US general population. The NSDUH data collection protocol was approved by the Institutional Review Board at RTI International.

The interview response rates according to the definition of the American Association for Public Opinion Research [21] were 76.0% in 2005 and 66.6% in 2018. The public access dataset used in this study included data on 553,893 adult participants (aged 18 years old or older) across the 2005–2018 surveys. The study sample was further limited to 11,732 adult participants who met the criteria for a drug use disorder (see below), had employer sponsored or individually purchased private health insurance, and responded to questions about coverage of drug use treatment under their health insurance plan.

### Measures

*Health insurance type* was assessed with a series of questions about the most common types of insurance. Each question included a preamble that provided a brief description of that type of health insurance. The preamble for private health insurance question was:

“Private health insurance can be obtained through work, such as through an employer, union, or professional association, or by paying premiums directly to a health insurance company. It includes coverage by a health maintenance organization (HMO), fee for service plans, and single service plans.”

Participants were next asked if they were currently covered by private health insurance. The next question asked whether participants obtained their private health insurance through their own work or their family members’ work, including through employers, unions, or professional associations.

*Coverage of drug use treatment* was assessed by asking privately insured participants if their insurance included coverage for treatment of “drug abuse”. Response options included: “yes,” “no,” and “I don’t know.” Only a small proportion of privately insured adults who met the criteria for a drug use disorder (see below) did not give one of these three responses (n = 80, 0.6%) and these participants were not included in the analysis. Separate questions were asked about coverage of treatment of “alcohol abuse or alcoholism” and “mental or emotional problems,” as well. This study focuses on coverage of drug use treatment.

*Drug use disorder* in the past year was defined by meeting criteria for abuse or dependence on heroin, pain relievers, hallucinogens, inhalants, tranquilizers, cocaine, stimulants, sedatives or marijuana ascertained using structured interviews based on the *Diagnostic and Statistical Manual of Mental Disorders*, *fourth edition* (DSM-IV) [22]. Reliability and validity of these assessments have been previously established [23,24]. Alcohol use disorder was similarly defined based on DSM-IV criteria for abuse or dependence using a structured interview.

*Drug use treatment* was ascertained by asking if participants had received any “treatment or counseling” for their drug use alone or in conjunction with treatment for alcohol use in the past 12 months. Treatment settings examined included only those generally covered by health insurance (i.e., inpatient, residential rehabilitation facility, outpatient specialty setting, mental health center, emergency room, doctor’s office) [25].

Information was also collected on participants’ sex, age, race/ethnicity, marital status, education, employment status, and annual family income compared to the federal poverty level (FPL).

### Statistical analysis

Analyses were conducted in four stages. First, changes in knowledge of coverage of drug use treatment (“know” vs. “don’t know”) and in coverage among those who knew about their coverage status (“covered” vs. “not covered”) were compared between the 2005–2013 (pre-ACA) period and the 2014–2017 (post-ACA) period. For these analyses, binary logistic regression models were used with a dummy variable for time period as the primary predictor of interest.

Second, demographic and drug use characteristics were compared between individuals with and without coverage among participants who knew their coverage status. Data were collapsed across all the survey years and comparisons were performed with contingency table analysis and design-based F-tests.

Third, among those who knew their coverage status, multivariable logistic regression models were used to assess the association of drug use treatment coverage with actual treatment use across all survey years. These models were adjusted for sex, age, race/ethnicity, income, employment, education, whether insurance was obtained through work or not, specific types of drug use disorder (heroin, cocaine, marijuana) and alcohol use disorder. In addition to examining the association of insurance coverage with any drug use treatment, associations with treatment in specific settings were examined.

A large proportion of adults reported not knowing their drug use treatment coverage status. To assess the sensitivity of study results to this large volume of missing data, two sets of sensitivity analyses were conducted. In the first set, it was assumed that among respondents who endorsed “don’t know,” all individuals who received treatment had coverage. Those who had not received treatment were randomly assigned coverage status so that the overall probability of coverage in the “don’t know” sample ranged from 10% to 90% (by 10 percentage point increments). In the second set of sensitivity analyses, no relationship between coverage and treatment was assumed. Thus, from 10% to 90% of the participants (by 10 percentage point increments) were randomly assigned coverage, without regard to their treatment status. For each set of sensitivity analyses, 100 data sets were simulated (a total of 1800 datasets). These simulated subsamples were each combined with the data from participants with known coverage status. The regression analysese were then repeated for each dataset and combined to produce an average odds ratio estimate with corresponding 95% confidence interval for the association of insurance coverage with use of services for every 10% increment in coverage. In a further step, analyses of individuals who did not know their drug use treatment coverage were repeated for two extreme scenarios; first assuming that *none* of these individuals had such coverage and then, assuming that *all* of them had such coverage. Overall, these simulations comprise the whole range of possible values for the prevalence of drug use treatment coverage (from 0% to 100%) and plausible associations between such coverage and actual receipt of treatment among participants who did not know their coverage status.

All analyses were conducted using STATA 16 (College Station, TX, 2019). Analyses accounted for the complex survey design and sampling weights of NSDUH. All reported percentages were weighted by survey weights to provide US population estimates. Thus, the percentages reported may not equal the percentages calculated based on the raw data. The weighted population estimates were divided by number of the survey years included (14) to provide an average annual estimate for the 2005–2018 period.

## Results

Of the 11,732 privately insured participants who met criteria for a drug use disorder between 2005 to 2018 and responded to questions about drug use treatment coverage, 6,808 (62.4%) knew about their insurance coverage for drug use treatment and 4,924 (37.6%) did not. Among those who knew whether or not they had coverage, 4,776 (75.1% of those who knew their coverage status) reported having such coverage and 2,032 (24.9%) reported not having coverage.

### Temporal trends of knowledge about drug use treatment coverage and having coverage

The proportion of privately insured participants with drug use disorder who knew about their drug use treatment coverage did not change appreciably between the pre-ACA (2005–2013) and post-ACA (2014–2018) periods: 62.6% knew their coverage in the 2005–2013 period compared to 62.4% in the 2014–2018 period (odds ratio [OR] = 0.98, 95% confidence interval [CI] = 0.87–1.10, *p* = .747). Among participants who knew about their coverage status, the proportion who reported having coverage modestly increased (from 73.5% in 2005–2013 to 77.5% in 2014–2018; OR = 1.24, 95% CI = 1.04–1.48, *p* = .015).

### Characteristics of participants with and without coverage

Table 1 presents the characteristics of those with and without drug use treatment coverage among participants who knew about their coverage status. Participants with coverage were older than those without coverage (60.8% vs. 43.7%, respectively, were 26+ years old, *p* < .001). A larger proportion of participants with coverage were from the non-Hispanic white racial/ethnic group (72.4% vs. 63.2%, *p* < .001). Furthermore, participants with coverage tended to have more education than those without coverage (58.7% vs. 50.4% had any college education, *p* < .001) and to have a higher family income (77.7% vs. 61.6% had incomes of 200% or more compared to FPL, *p* < .001) (Table 1). Covered participants were less likely than those without coverage to meet criteria for marijuana use disorder (55.2% vs. 61.3%, *p* = .014). However, the two groups did not differ regarding other substance use disorders. Participants with coverage for drug use treatment were more likely to have obtained their insurance through employment (92.0% vs. 81.8%, *p* < .001) (Table 1).

**Table 1:** Characteristics of 6,808 privately insured adults with drug use disorders with and without coverage of drug use treatments in the National Survey on Drug Use and Health, 2005–2018.

Variable	Total n = 6,808		With coverage n = 4,776 (75.1%)		Without coverage n = 2,032 (24.9%)		Comparison of participants with and without coverage
	Average n in 1000s^a^	%^b^	Average n in 1000s^a^	%^b^	Average n in 1000s^a^	%^b^	Design-based F test, *p*
Sex							
Female	601	33.9	456	34.3	145	32.9	
Male	1,170	66.1	874	65.7	296	67.1	.41, .525
Age, years							
18–25	769	43.4	521	39.2	249	56.3	
26–34	372	21.0	280	21.1	92	20.7	
35–49	397	22.4	334	25.1	63	14.2	
50+	233	13.2	194	14.7	38	8.7	17.82, < .001
Race/ethnicity							
Non-Hispanic white	1,242	70.1	963	72.4	279	63.2	
Non-Hispanic black	213	12.0	151	11.4	62	14.0	
Hispanic	219	12.4	148	11.1	71	16.1	
Other	98	5.5	68	5.1	30	6.8	7.30, < .001
Education							
<12 years	215	12.2	147	11.0	69	15.6	
12 years	553	31.2	403	30.3	150	34.0	
Some college	623	35.1	476	35.8	146	33.1	
College graduate	381	21.5	304	22.9	76	17.3	6.32, < .001
Employment							
Working	1,371	77.5	1,042	78.5	328	74.6	
Student/homemaker	116	6.5	78	5.9	38	8.5	
Unemployed	198	11.2	141	10.6	57	12.9	
Retired	37	2.1	30	2.3	7	1.5	
Disabled	48	2.7	36	2.7	11	2.5	1.86, .136
Income, compared to FPL							
<100%	185	10.6	107	8.1	78	18.1	
100%-<200%	274	15.7	187	14.2	87	20.2	
200% +	1,289	73.7	1,023	77.7	266	61.6	43.86, < .001
Heroin use disorder	101	5.7	81	6.1	20	4.6	1.81, .181
Cocaine use disorder	258	14.5	200	15.0	58	13.0	1.75, .188
Marijuana use disorder	1,005	56.7	734	55.2	270	61.3	6.29, .014
Alcohol use disorder	721	40.7	538	40.4	184	41.6	.29, .593
Insurance through employment	1,578	9.2	1,222	92.0	355	80.8	79.76, < .001

Data from the National Survey on Drug Use and Health, 2005–2018.

^a^ Average annual population estimate for the 2005–2018 period estimated based on survey weights.

^b^ All percentages are weighted by survey weights.

### Association of drug use treatment coverage with receiving treatment

Table 2 presents analyses of the association of having drug use treatment coverage with receiving treatment. Having coverage was significantly associated with receiving such treatment (adjusted odds ratio [aOR] = 2.09, 95% CI = 1.61–2.72, *p* < .001). Nevertheless, even among those with coverage, only 13.4% received treatment in the past year (Table 2).

**Table 2:** Drug use treatment and treatment setting among privately insured adults with drug use disorders with and without coverage of drug use treatments in the National Survey on Drug Use and Health, 2005–2018.

Variable	Total	With coverage N = 4,385 (75.0%)	Without coverage N = 1,896 (25.0%)	Comparison of participants with and without coverage
	%^a^	% ^a^	% ^a^	OR (95% CI), *p*^*b*^
Any drug use treatment	11.8	13.4	7.2	2.09 (1.61–2.72), < .001
Drug use treatment setting				
Inpatient	4.0	4.7	2.2	2.34 (1.42–3.85), .001
Treatment in a residential				
rehabilitation facility	5.0	5.7	2.9	2.00 (1.26–3.19), .004
Outpatient specialty treatment	6.6	7.5	4.0	2.05 (1.45–2.90), < .001
Mental health center	4.4	5.1	2.5	2.08 (1.39–3.10), < .001
Emergency room	2.3	2.7	1.0	3.18 (1.52–6.64), .002
A doctor’s office	4.4	5.1	2.3	2.16 (1.34–3.34), .002

^a^ All percentages are weighted by survey weights.

^b^ Analyses adjusted for sex, age, race/ethnicity, income, employment, education, whether insurance was obtained through work or not, and heroin, cocaine, marijuana and alcohol use disorders.

**Fig 1:**
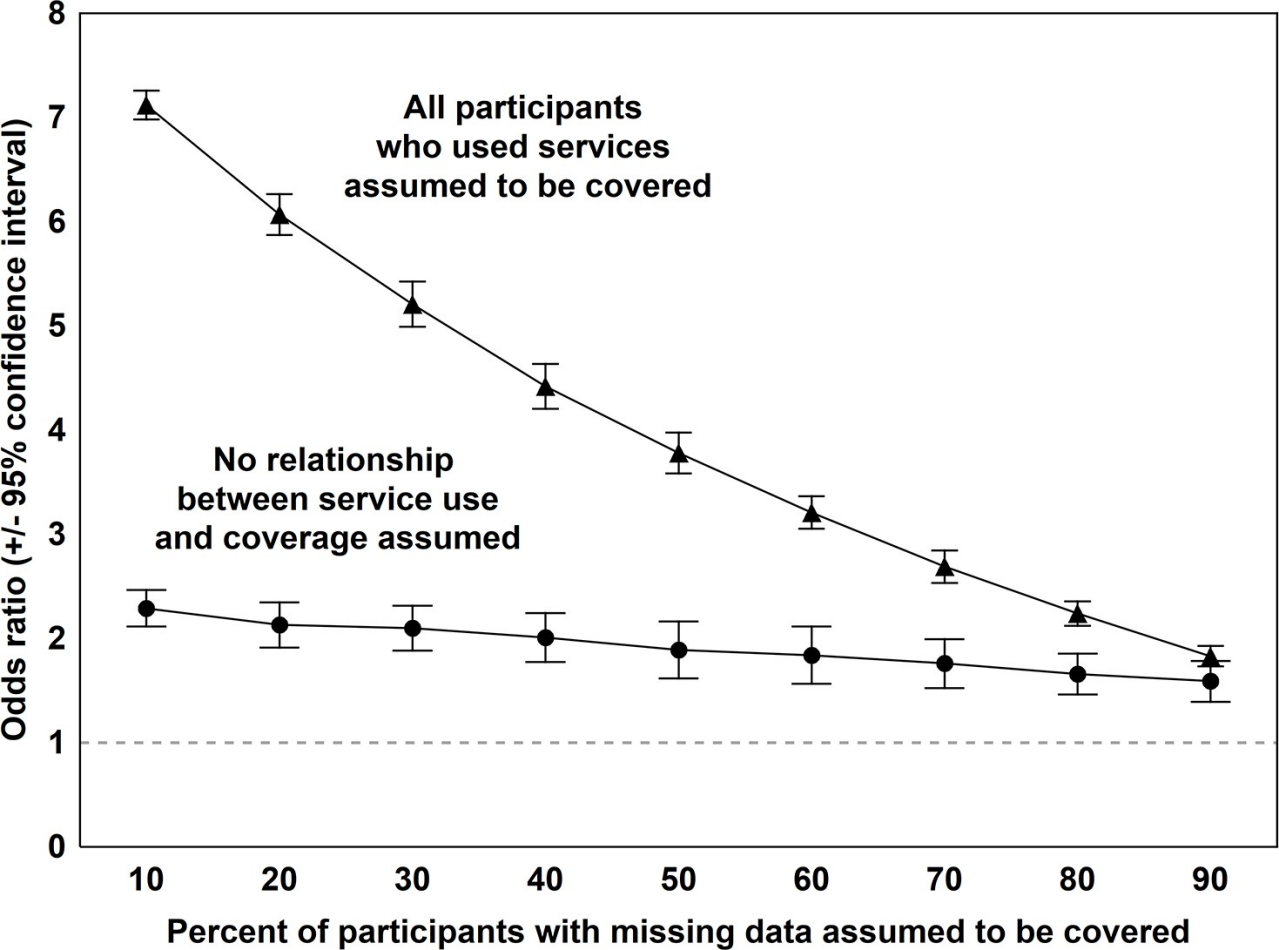
Results of logistic regression analyses of the association of drug use treatment coverage with actual receipt of treatment among privately insured participants with substance use disorders in the National Survey of Drug Use and Health, 2005–2018. The odds ratios are based on simulations assuming different levels of coverage and different degrees of association of coverage with receipt of treatment among participants who did not know their coverage status. Each calculated odds ratio is based on 100 simulations.

In the sensitivity analyses in which coverage and the association of coverage with drug use treatment were simulated for participants who reported not knowing their coverage status, the association of coverage with actual use of treatment services remained significant with odds ratios >1 for a broad range of simulated values for coverage (10% to 90% of the privately insured who did not know their coverage status assumed to have coverage) and association of coverage with treatment (from no relationship between coverage and treatment to the scenario in which all those who received treatment were assumed to have drug use treatment coverage) (Fig 1). In addition, two extreme scenarios were envisioned for participants who did not know their coverage status. In the first scenario, none of these participants were assumed to have coverage and, in the second scenario, all of these participants were assumed to have coverage. The odds ratios for the association of drug treatment coverage with actual receipt of treatment remained significant in both of these extreme scenarios (OR = 2.46, 95% CI = 1.91–3.16, *p* < .001 and OR = 1.46, 95% CI = 1.14–1.88, *p* = .004, respectively).

## Discussion

The proportion of privately insured adults who reported having drug use treatment coverage modestly increased following implementation of the ACA. Nevertheless, a sizable share of privately insured adults with drug use disorders continue to lack coverage for drug use treatment. Estimating the size of this group based on the NSDUH survey is challenging because over a third of participants reported not knowing whether their health plan offered coverage for drug treatments. Among those who knew, individuals without coverage were typically younger, more likely to be from minority groups, to have lower income and to have less education. These indicators of social adversity are associated with a greater burden of drug use disorder and unmet need for treatment [26–28].

It is concerning that a substantial proportion of privately insured individuals with drug use disorders appear not to know whether their insurance plans cover treatment of drug use. Lack of knowledge about services is often considered a barrier to behavioral health service use [29–31]. Lack of knowledge about benefits is also known to act as a barrier to accessing other health services, such as preventive reproductive health services [32]. Adults with drug use disorders who have not sought treatment and do not know whether their plans cover drug use treatment may have a low level of treatment seeking intention [33] or be in the earliest “precontemplation” stage [34] of readiness to seek treatment.

MHPAEA and ACA both attempted to improve coverage of substance use treatments under private insurance plans by requiring parity in coverage of these services. Yet despite their remarkable progress, they may not have been fully successful in removing financial barriers to these services [35]. A recent commentator compared the accomplishment of MHPAEA and ACA to a glass that is “partially empty” [35]. Many of the participants without drug use treatment coverage in this study may have been covered by grandfathered plans that were exempted from the essential health benefits requirement of ACA. The continued presence of these plans in the private insurance market may impede treatment provision to thousands of US adults with drug use disorders.

Another challenge on the way to full implementation of parity is the difficulty in monitoring non-quantitative limits on treatments (NQLTs) imposed by some plans, including preauthorization requirements and other utilization management processes as well as formulary designs [6,35,36]. It is conceivable that some of the participants in this study may have reported not having insurance coverage of drug use treatment because treatments they received were not covered as a result of these NQLTs. Other data suggest that plans offered in Marketplaces were more likely to employ narrow and tiered behavioral health provider networks [37–39], thereby reducing access to services.

There are fears that some of the progress toward mental health and subtance use treatment parity may be rolled back in the future [40–42]. Along with other provisions of ACA, the essential health benefit requirement faces challenges in the current policy environment. In particular, the introduction of Association Health Plans (AHP) and short-term health plans, which are exempt from the essential health benefit requirement, may erode recent expansions of mental health and substance use treatment coverage [40,41]. Although the full impact of these new changes is not yet known [43], their development likely increases variation and uneveness in coverage of mental health and substance use services across the private health insurance market.

The results of this study were different from the results of an earlier study based on 2005–2009 NSDUH that also examined the association of private insurance coverage of drug use treatment with actual use of treatmen services [16]. That study found that 19.8% of those with coverage vs. 6.7% of those without coverage used services. However, treatment services in that study included alcohol and/or drug use treatment; whereas, the present study examined only drug use treatment.

Several limitations of this study should be considered. First, drug use treatment coverage was assessed by self report which is open to recall and social desirability biases. Second, drug use treatment coverage was based on a single question. Coverage for drug use treatment may vary according to the type of services (e.g., inpatient treatment, outpatient services) [44]. Third, knowledge about insurance coverage for drug use treatment may be a direct consequence of seeking drug use treatment. However, this factor does not impact the main findings of the study, because the primary analysis of the association between coverage and treatment was limited to individuals with a knowledge of their coverage. Fourth, NSDUH does not assess continuity, volume, quality and effectiveness of treatment that may also be influenced by insurance coverage. Finally, NSDUH did not identify specific evidence-based treatments of drug use disorder, such as agonist therapies for opioid use disorders, nor did the survey assess continuity in treatment or quality of care. The association of insurance coverage with these important characteristics of treatment needs to be assessed in future research.

## Conclusion

The findings of this study highlight the role of private insurance coverage in treatment seeking for drug use disorders. The results underscore the importance of renewed efforts to preserve and build on the essential health benefit’s mandate requiring drug treatment benefits under private health insurance plans and the potential threat posed by counter initiatives aimed at undermining this essential requirement of the ACA [40]. While expansion of insurance coverage would be expected to increase service use, low treatment rates among insured individuals underscore the presence of other barriers to service use among covered individuals, such as narrower provider networks [37–39] and NQLTs imposed by some plans [6,35,36]. These provider-side barriers blunt the effect of insurance coverage expansion and parity laws and call for further policy reforms to ensure adequate access to services for individuals in need of drug use treatments.
